# A “Mesoporous Oxygen Chamber” Scaffold with Antibacterial and Early Immunomodulatory Effect for Promoting Bone Regeneration

**DOI:** 10.1002/advs.202506737

**Published:** 2025-09-16

**Authors:** You Fu, Dan Lin, Zhicen Lu, Jian Wang, Jing Zhao, Zhiyuan Zhang, Bing Fang, Xiao Yang

**Affiliations:** ^1^ Department of Orthodontics Shanghai Ninth People's Hospital, Shanghai Jiao Tong University School of Medicine, College of Stomatology, Shanghai Jiao Tong University, National Center for Stomatology, National Clinical Research Center for Oral Diseases, Shanghai Key Laboratory of Stomatology & Shanghai Research Institute of Stomatology Research Unit of Oral and Maxillofacial Regenerative Medicine Chinese Academy of Medical Sciences Shanghai 200011 China; ^2^ Shanghai University of Medicine and Health Sciences Shanghai 201318 China; ^3^ Fujian Key Laboratory of Oral Diseases & Fujian Provincial Engineering Research Center of Oral Biomaterial & Stomatological Key Laboratory of Fujian College and University, School and Hospital of Stomatology Fujian Medical University Fuzhou 350122 China; ^4^ Department of General Dentistry Shanghai Ninth People's Hospital, Shanghai Jiao Tong University School of Medicine College of Stomatology, Shanghai Jiao Tong University, National Center for Stomatology, National Clinical Research Center for Oral Diseases Shanghai Key Laboratory of Stomatology & Shanghai Research Institute of Stomatology Research Unit of Oral and Maxillofacial Regenerative Medicine Chinese Academy of Medical Sciences Shanghai 200011 China; ^5^ Shanghai Dingchang Labor Security Consulting Co., Ltd. Shanghai 200011 China; ^6^ Department of Oral and Maxillofacial‐Head Neck Oncology Shanghai Ninth People's Hospital, Shanghai Jiao Tong University School of Medicine College of Stomatology National Center for Stomatology National Clinical Research Center for Oral Diseases Shanghai Key Laboratory of Stomatology & Shanghai Research Institute of Stomatology Research Unit of Oral and Maxillofacial Regenerative Medicine Chinese Academy of Medical Sciences Shanghai 200011 China; ^7^ Department of Oral & Cranio‐Maxillofacial Surgery Shanghai Ninth People's Hospital, Shanghai Jiao Tong University School of Medicine College of Stomatology, Shanghai Jiao Tong University, National Center for Stomatology, National Clinical Research Center for Oral Diseases, Shanghai Key Laboratory of Stomatology & Shanghai Research Institute of Stomatology Research Unit of Oral and Maxillofacial Regenerative Medicine Chinese Academy of Medical Sciences Shanghai 200011 China

**Keywords:** antimicrobial peptide, Mesoporous bioactive glass, osteoimmunomodulatory, oxygen therapy

## Abstract

Regeneration of bone defects are frequently hindered by severe inflammation, immunogenicity after bone repairing material implantation, and microbial infections. Oxygen therapy is reported to downregulate the levels of pro‐inflammatory cytokines and proteases, alleviating inflammation and promoting regeneration. Mesoporous bioactive glass (MBG) is marked by its osteoinductivity and mesoporous structure for drug delivery. In this study, an oxygen‐loaded and antimicrobial peptide (AP)‐functionalized scaffold (MBGAPO) is synthesized and proven with sufficient oxygen‐delivery capacity of mesopores, promising antibacterial ability against *E.coli* and *MRSA*, multiplex‐immunomodulatory effects, and potent osteoinductivity both in vitro and in vivo. In cranial defect models of mouse and rat, MBGAPO created a mild immune microenvironment that accelerated inflammation alleviation and facilitated immune cells transformation toward anti‐inflammatory phenotypes in the initial stage of bone regeneration, and exhibited a superior immune modulatory effect than Bio‐oss (an FDA‐approved bone substitute). In vivo results indicated that oxygen delivery promoted bone regeneration within the scaffold, and AP functionalization facilitated the bridging of surrounding tissue in the defect area. In summary, mesoporosity‐based oxygen delivery is first proven as a promising osteoimmunology therapeutic strategy, and its combination with antimicrobial peptides can be extended to more regenerative and disease treatment applications that may arouse broader interests of researchers.

## Graphical abstract

In this study, an oxygen‐loaded and antimicrobial peptide (AP)‐functionalized scaffold (MBGAPO) is synthesized. With mesoporous structure of MBG applied as an “oxygen storage,” and AP‐provided antibacterial ability against *E.coli* and *MRSA*. MBGAPO exhibited osteogenic and multiplex‐immunomodulatory effects. MBGAPO significantly increased neutrophil and macrophage infiltration, accelerated inflammation alleviation, and facilitated immune cells transformation toward anti‐inflammatory phenotypes.

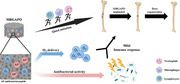

## 1 Introduction

Bone defects result from trauma, infection, tumors, surgical debridement of osteomyelitis, and various congenital diseases and can lead to pain, dysfunction, deformity, and infection, which seriously affects the life quality of patients.^[^ ^1^, ^2^, ^3^ ^]^ Due to its long treatment time and high surgical risks, bone defect treatment remains one of the great challenges in clinical.^[^ ^4^ ^]^

Though bone grafts and bioactive scaffolds, as regular treatment for bone defects, are reported to facilitate bone repair, they also increase the risk of infection and inflammation.^[^ ^5^ ^]^ Infection and inflammation are reported to prolong immunoreactions and impair bone regeneration,^[^ ^6^, ^7^ ^]^ therefore it is critical to create a suitable immune microenvironment around the implanted materials in bone defects. Bone regeneration involves various immune cells, cytokines, chemokines, and complements ^[^ ^8^, ^9^ ^]^ among which macrophages are closely related to inflammation regulation and pathogen clearance^[^ ^10^, ^11^ ^]^ and other immune cells like neutrophils and CD4^+^ T cells also play indispensable roles.^[^ ^12^, ^13^ ^]^ Comprehensively understanding the immune microenvironment around the implantation is conducive to improving the biomaterial design for bone defect treatment.

Oxygen therapy (OT) is widely used in various diseases of the cardiovascular system, autoimmune diseases, infection, etc.^[^ ^14^ ^]^ OT has been applied to treat bone defects in recent years,^[^ ^15^, ^16^ ^]^ and proven as an effective strategy of regulating cell metabolic reprogramming, osteointegration, and angiogenesis of scaffolds,^[^ ^16^, ^17^ ^]^ as well as alleviating inflammation to facilitate bone regeneration.^[^ ^18^ ^]^ Oxygen‐generating compounds, including CaO_2_ and H_2_O_2_ are incorporated into biomaterials for OT,^[^ ^19^, ^20^, ^21^ ^]^ however, peroxides raise the risks of generating superfluous oxygen and reactive oxygen species (ROS), which disrupt tissue regeneration.^[^ ^22^ ^]^ Design of novel bioactive materials that deliver oxygen without generating detrimental by‐products is important for the development of OT in bone defect treatment.

Mesoporous bioactive glass (MBG) is one of the best candidates for bone‐repairing scaffolds due to its biocompatibility, osteoinductivity, biodegradability, and other positive biological effects.^[^ ^23^, ^24^, ^25^, ^26^, ^27^ ^]^ The high specific surface area, large pore volume, and adjustable mesoporous structure^[^ ^28^, ^29^, ^30^ ^]^ endow MBG with excellent loading capacity and delivery of drugs and biofunctional agents.^[^ ^31^, ^32^ ^]^ In recent years, researchers have begun the exploration of gas storage and delivery by mesoporous materials in biomedical applications, including nitric oxide (NO) in cancer therapy,^[^ ^33^, ^34^ ^]^ and H_2_ for remodeling senescence microenvironment and improving bone regeneration.^[^ ^35^ ^]^ So far, few attempts have been made to apply MBG as oxygen storage and delivery in bone regenerative therapy.

In this study, an oxygen‐loaded and antimicrobial peptide (AP)‐functionalized scaffold (MBGAPO) was synthesized. The mesoporous structure of MBG was first used as a “oxygen storage” instead of a “drug capsule” for therapeutic use. Biosafety, oxygen‐delivering capacity, and antibacterial effects of MBGAPO were evaluated, and its osteogenic performance was validated in vitro and in vivo. In addition, inflammatory state and infiltrating immune cells were also systematically investigated for a comprehensive understanding of the immune microenvironment created by MBGAPO.

## 2 Results and Discussion

### 2.1 Preparation and Characterization of Scaffolds

**Figure 1:**
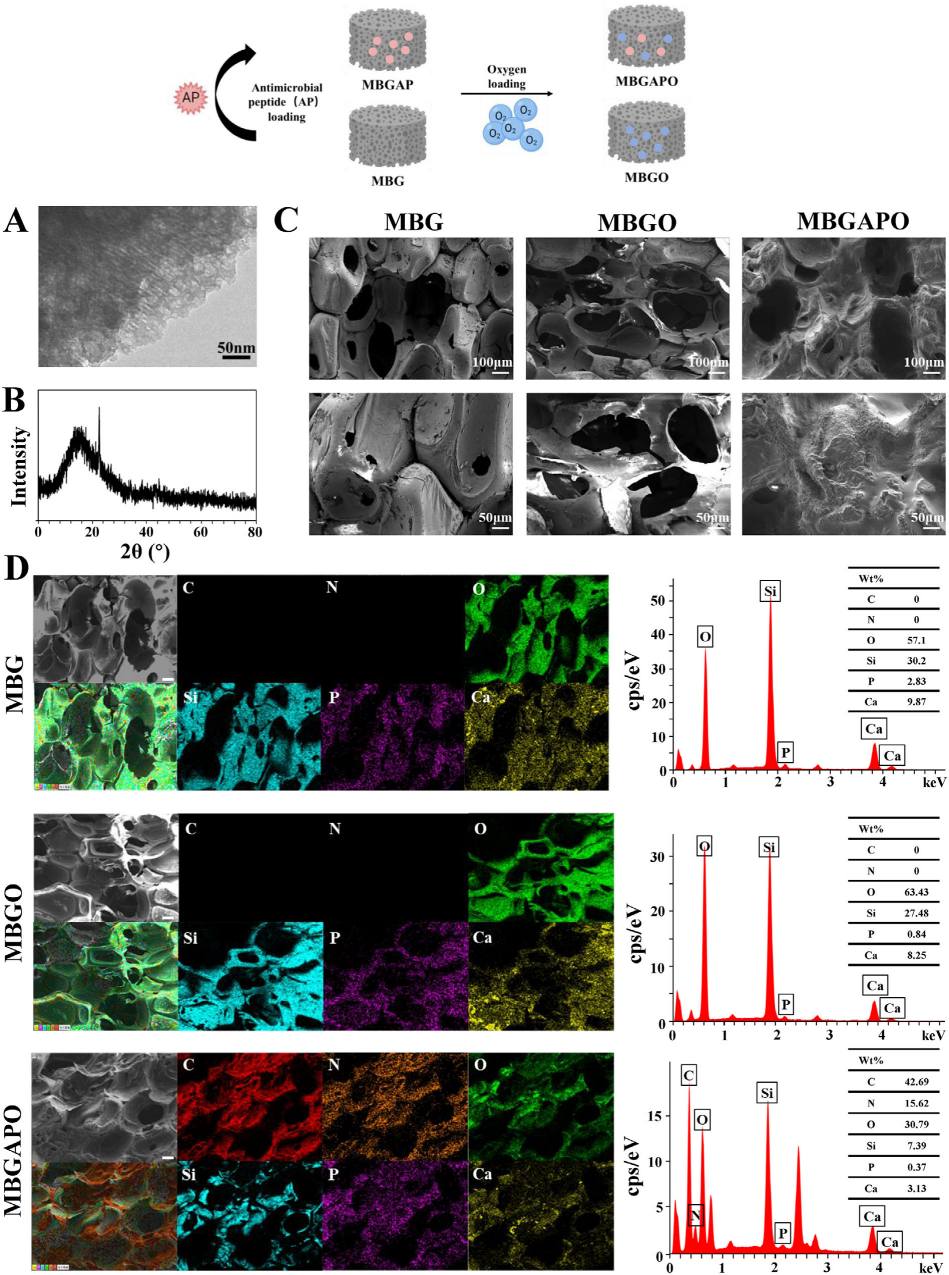
Characterizations of MBG/MBGO/MBGAPO scaffolds. A) TEM images of MBG scaffolds. B) XRD pattern of MBG scaffolds. C) SEM images of MBG/MBGO/MBGAPO scaffolds. D) Element mapping including C, N, O, Si, P and Ca and EDS spectrum of MBG/MBGO/MBGAPO scaffolds. Scale bar = 100 µm.

The mesoporous structure and phase composition of MBG were identified via transmission electron microscope (TEM) and wide‐angle X‐ray diffraction (XRD). Before and after antimicrobial peptide functionalization and O_2_ loading, the surface morphology of MBG/MBGO/MBGAPO scaffolds was observed via scanning electron microscope (SEM). Mesoporous tunnels (pore diameter 6–8 nm) formed via F127 (mesoporous template) self‐assembly were observed in MBG in TEM images (**Figure** **1**A). Wide‐angle X‐ray diffraction (XRD) pattern of MBG demonstrated the phase composition of MBG as amorphous silicon dioxide (Figure 1B). As shown in SEM images (Figure 1C), all scaffolds exhibited similar macroporous structure (pore size: 200–500 µm, Figure S2, Supporting Information), which was duplicated from the polyurethane sponge macroporous template. Macrporous and mesoporous structures were not altered after antimicrobial peptide (AP) functionalization and O_2_ loading, whereas AP functionalization roughened the surface morphology of the scaffold: MBG and MBGO exhibited a similar smooth surface, and MBGAPO scaffold showed a rougher surface (Figure 1C). To achieve deep understanding of the elements distribution on MBG/MBGO/MBGAPO scaffolds, energy spectrum analysis (EDS) mapping and element distribution analysis were performed. The EDS results confirmed Si, Ca P, and O in all groups, as the main composition of the Si/Ca/P trinary oxide (Figure 1D); C and N elements in MBGAPO group indicated successful loading of antimicrobial peptide (Figure 1D). Since SEM and EDS scanning are carried out under vacuum conditions, alteration of oxygen element distribution before and after O_2_ loading cannot be detected theoretically. However, the EDS mapping of MBGO still showed a slightly higher oxygen ratio than MBG (Figure 1D).

### 2.2 O_2_ Release Capacity, Cytocompatibility, and Antibacterial Effects of MBGAPO

**Figure 2:**
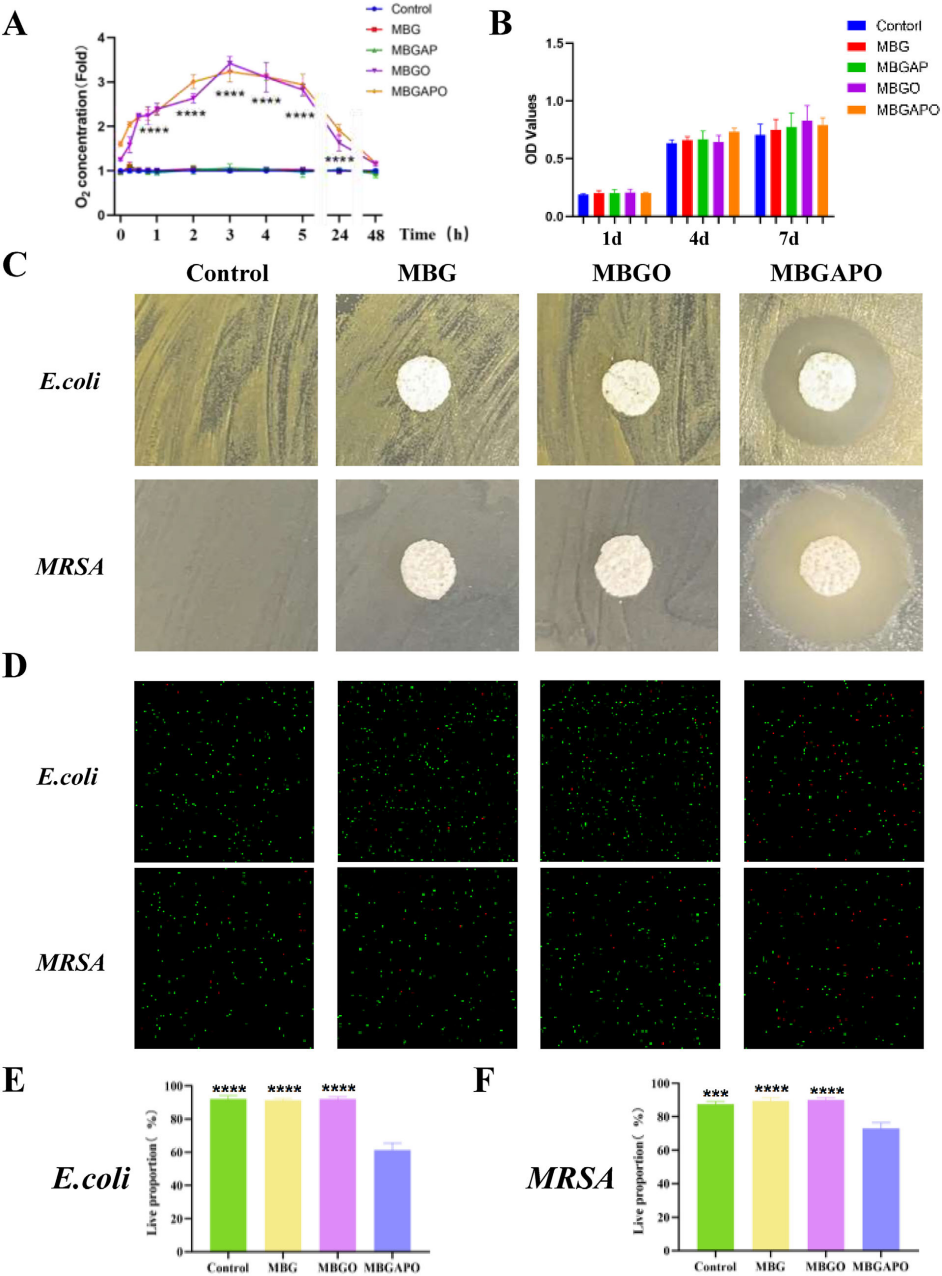
Oxygen releasing capacity, biosafety and antibacterial ability of MBGAPO scaffolds. A) Dissolved oxygen measurement in ddH_2_O in the presence of different scaffolds under physiological conditions (*n* = 3). B) Effects of different scaffolds on cell viability of BMSCs. C) Antibacterial effects of MBGAPO on Escherichia coli (*E.coli*) and Methicillin‐resistant Staphylococcus aureus (*MRSA*) using inhibition zone method. D) Representative images of live/dead bacterial staining of *E.coli* and *MRSA* with different treatment. E,F) Live proportions of *E.coli* and *MRSA* with different treatment. Bars The bars show the mean ± SD. ^***^represents *p *< 0.001, ^****^represents *p* < 0.0001. (*n* = 3).

To identify the O_2_ delivery behavior of scaffolds, the alteration of dissolved oxygen in ddH_2_O after scaffold immersing was evaluated (**Figure** **2**A). Without oxygen loading, MBG and MBGAP scaffolds exhibited no oxygen release, while MBGO and MBGAPO scaffolds exerted continuous oxygen release within 48 h, reaching a highest threefold local O_2_ concentration and maintaining an over 2‐fold O_2_ concentration within 24 h (Figure 2A).

CCK8 assay showed that all scaffolds exhibited excellent biosafety and cytocompatibility, with no significant toxicity to BMSCs (Figure 2B). Antibacterial effects of scaffolds were detected by the inhibition zone method and bacteria live/dead staining of *Escherichia coli (E.coli)* and *Methicillin‐resistant Staphylococcus aureus (MRSA)*. Without an antibacterial peptide, no obvious zone of inhibition was observed in the control/MBG/MBGO group, whereas a distinct zone of inhibition was exhibited around MBGAPO scaffold (Figure 2C). The result of bacteria live/dead staining indicated that MBGAPO scaffold significantly reduced the live ratio of bacteria compared to other groups (Figure 2D–F). These results confirmed the potent antibacterial effect of MBGAPO against *E.coli* and *MRSA*.

### 2.3 In Vitro Osteogenic Differentiation of BMSCs on MBGAPO

**Figure 3:**
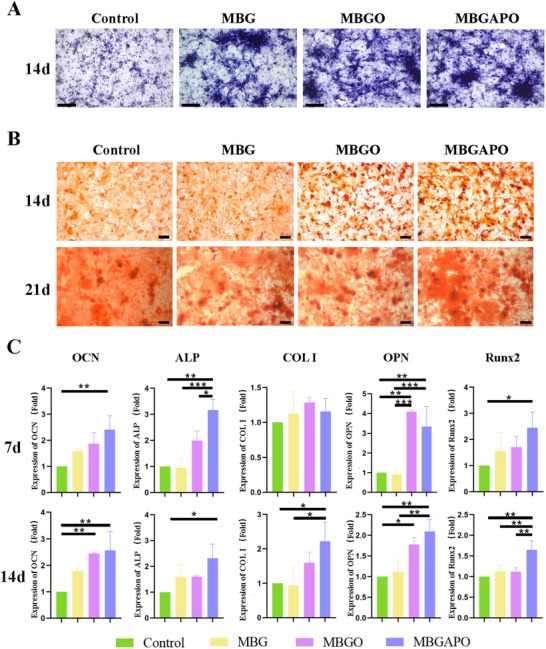
Effects on osteogenic differentiation of MBGAPO in vitro. A) Repressive images of ALP staining of co‐cultured BMSCs at day 14. Scale bar= 500 µm. B) Repressive images of alizarin red staining of co‐cultured BMSCs at day 14 and day 21. Scale bar= 200 µm. C) Osteogenesis‐related gene mRNA expression levels of co‐cultured BMSCs at day 7 and day 14. Bars The bars show the mean ± SD. ^*^ represents *p* < 0.05, ^**^ represents *p* < 0.01, ***represents *p *< 0.001. (*n* = 3).

To evaluate the osteogenic differentiation of BMSCs on MBGAPO, alkaline phosphatase (ALP) staining and alizarin red staining were performed. ALP is the hallmark enzyme of mature osteoblasts, and calcium nodules formed by osteoblasts are also a marker of osteoblasts. Results of ALP staining showed that positive staining areas in MBG, MBGO, and MBGAPO groups were significantly increased than the control group at day 14 (**Figure** **3**A), indicating the excellent osteoinductivity of MBG. Alizarin red staining demonstrated significantly increased calcium depositions in MBGAPO and MBGO groups at day 14, and the highest amount of calcium nodules was found in MBGAPO group at day 21 (Figure 3B). These results indicated that oxygen delivery and AP functionalization enhanced the osteoinductivity of MBG.

To better demonstrate the osteogenic‐inducing properties of the scaffolds, bone‐related gene expressions were analyzed. Osteocalcin (OCN) occurs at the advanced stage of osteoblast differentiation and binds to Ca^2+^ to regulate calcium homeostasis and bone mineralization. The expression of OCN was higher in the MBGAPO group than in the control group both at day 7 and day 14 (Figure 3C). In addition, the expression of OCN in the MBG group exhibited a statistically significant disparity at day 14 when compared to the control group (Figure 3C). ALP is the most widely recognized marker of osteoblast activity. The expression of ALP was much higher in the MBGAPO group compared to the other three groups at day 7 (Figure 3C). It was also higher than that in the control group at day 14 (Figure 3C). Type I collagen (COL I) is an extracellular matrix protein that stimulates osteoblast adhesion and differentiation. The expression of COL I showed no significant difference among all groups at day 7, while the expression of COL I in the MBGAPO group was found to be higher compared to the control group and MBG group (Figure 3C). Osteopontin (OPN) is one of the abundant non‐collagen proteins in the bone matrix produced by osteoblasts and osteoclasts, which can effectively stimulate the osteoclast formation and absorption activity of mature osteoclasts. The expression of OPN in the MBGO group and MBGAPO group both showed a significant difference when compared to the control group and MBG group at day 7 (Figure 3C, OPN). At day 14, OPN expression in the MBGO group and MBGAPO group showed a significant difference compared to the control group (Figure 3C). In addition, OPN expression was higher in the MBGAPO than in the MBG group (Figure 3C). Runt‐related transcription factor 2 (Runx2) is the central control gene of osteoblast phenotype. The importance of Runx2 in osteoblast differentiation has been demonstrated. Destruction of one copy of the Runx2 gene leads to skull dysplasia, while knockout of the Runx2 gene in mice prevents osteoblast development. The expression of Runx2 in the MBGAPO group showed a statistic difference compared to the control group at day 7 and was higher than the other three groups at day 14 (Figure 3C). O_2_‐loading scaffolds (MBGO and MBGAPO) upregulated late osteogenesis markers OCN and OPN at day 14 compared to the control group. The expression level of OPN in the MBGO group was higher than that in the MBG group at day 7. MBGAPO upregulated early osteogenesis markers ALP, COL I, and Runx2, and late osteogenesis markers OCN and OPN at day 7 and day 14 (except COL I at day 7). These results identified that MBG had an osteoinductivity in vitro; MBGAPO and MBGO had a more potent effect on promoting osteogenic differentiation of BMSC in vitro. In consideration that AP had no osteoinductive property, the osteoinductivity of MBGAPO indicated that AP might promote BMSC adhesion and differentiation, regulate bone‐related gene expression, and indirectly promote osteogenesis.

### 2.4 In Vivo Bone Regeneration and Anti‐Inflammation Effect of MBGAPO

**Figure 4:**
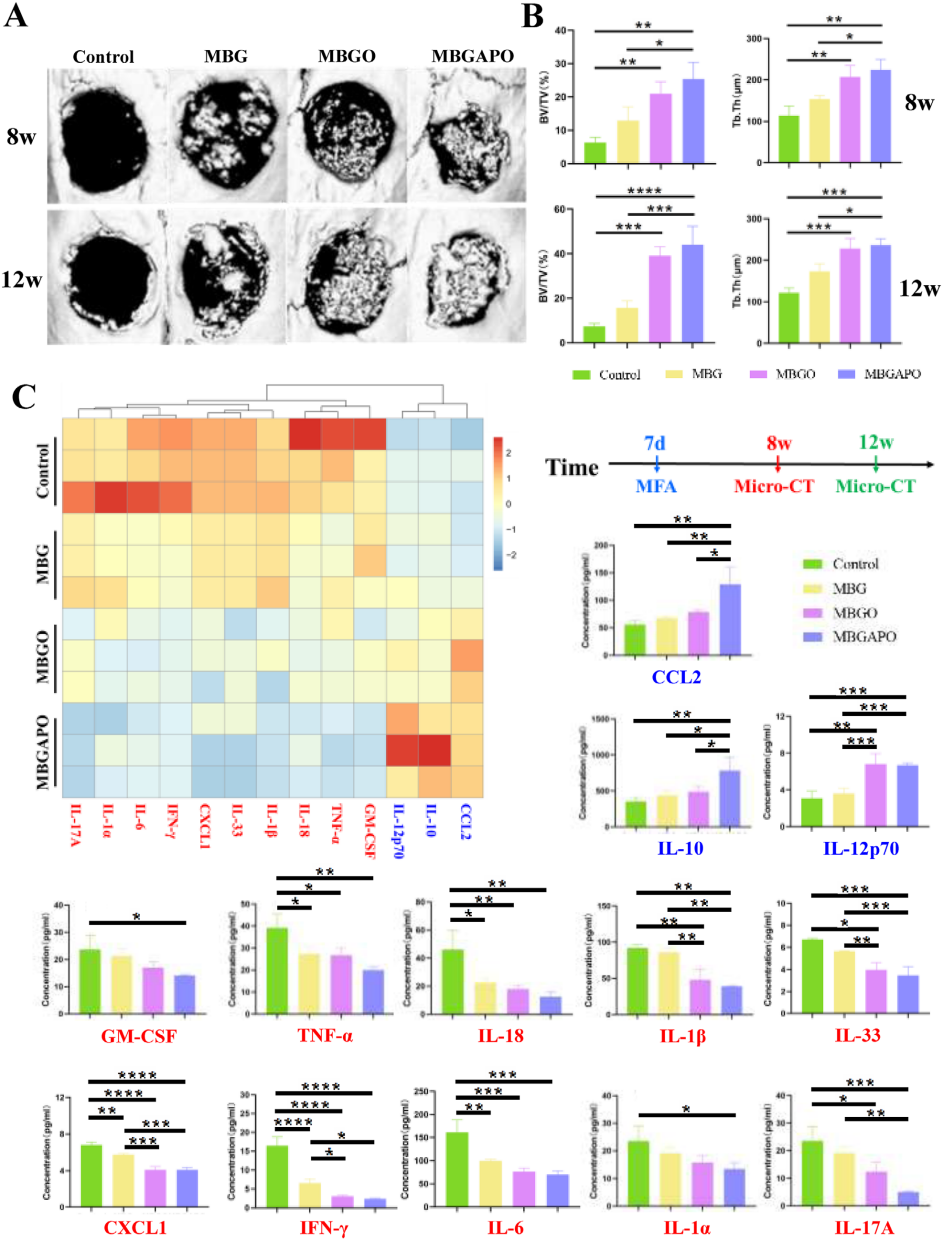
Osteogenic promotion function and anti‐inflammation effects of MBGAPO in vivo. A) Micro‐CT images and B) Micro‐CT quantification of Total bone volume/Total tissue volume (BV/TV) and Trabecular thickness (Tb.Th). C) Inflammation‐related cytokine secretion levels in the bone regeneration microenvironment (MFA: Multifactor analysis). Bars The bars show the mean ± SD. ^*^ represents *p* < 0.05, ^**^ represents *p* < 0.01, ^***^represents *p* < 0.001, ^****^represents *p* < 0.0001. (*n* = 3). Anti‐inflammatory cytokines: blue, pro‐inflammatory cytokines: red.

To further validate the effects of MBGAPO on bone regeneration in vivo, a cranial defect model was established. MBG/MBGO/MBGAPO scaffolds were implanted into the bone defect to induce bone regeneration. 8 weeks and 12 weeks after the implantation, samples were collected, and micro‐CT scanning was applied. It could be observed that the defect regions were better repaired in the MBGO and MBGAPO groups than other groups (**Figure** **4**A). Based on the micro‐CT reconstruction images, we also quantified the formation of the new bone. The results of total bone volume/total tissue volume (BV/TV) and trabecular thickness (Tb. Th) showed that there was superior bone formation in the MBGO group and MBGAPO group compared with the control group (Figure 4B). In the meantime, MBGAPO also showed stronger osteogenic activity than MBG (Figure 4B). In all, MBG showed a pro‐osteogenesis effect, MBGO significantly promoted bone regeneration within the scaffold; and MBGAPO not only promoted new bone formation on the scaffold, but also facilitated the growth of the surrounding tissue and bridging of the defect area (Figure 4A). Interestingly, though AP did not exhibit in vitro osteoinductivity, the superior in vivo bone repairing efficiency of MBGAPO might result from the antibacterial effect of AP, which lowered local immune response. Animal models mimicked the regenerative microenvironment in the human body and provided a more convincing and objective evaluation of the ability to induce osteogenesis of biomaterials. Our in vivo results demonstrated that MBGAPO had potent osteoinductive capacity, which was consistent with our in vitro results. In light of this, the MBGAPO scaffold could be a promising treatment strategy for bone defect patients.

To better investigate the inflammation state in the regenerative microenvironment, we applied a multifactor analysis to demonstrate the inflammation‐related cytokine levels at day 7 (the peak of the neutrophils and macrophages), including interleukins (IL‐1α, IL‐1β, IL‐6, IL‐10, IL‐12p70, IL‐17A, IL‐18, IL‐33), chemokines (CXCL1, CCL2), cell death‐related cytokines (IFN‐γ, TNF‐α), and colony stimulating factor (GM‐CSF). Results showed that there was a much milder inflammatory response in the microenvironment of the MBGAPO group than that other group (Figure 4C). It was demonstrated that MBG exhibited a slight anti‐inflammatory effect with downregulated TNF‐α, CXCL1, IFN‐γ, and IL‐18 expressions compared to the control group. O_2_‐loading scaffolds (MBGO and MBGAPO) exhibited potent anti‐inflammatory function by upregulated secretion levels of anti‐inflammatory cytokines such as CCL2, CXCL1, IL‐10, and IL‐12p70 (Figure 4C). In the meantime, pro‐inflammatory cytokines, including GM‐CSF, TNF‐α, IL‐18, IL‐1β, IL‐33, IFN‐γ, IL‐6, IL‐1α, and IL‐17A were obviously lower in the MBGAPO group (Figure 4C). Notably, MBGAPO and MBGO showed better anti‐inflammatory effects than MBG. In view of the fact that inflammation had a huge influence on bone regeneration, O_2_‐loading scaffolds could be a promising bioactive scaffold candidate for bone defects. Interestingly, the expression level of CCL2 and IL‐6 was higher in the MBGAPO group than that in the MBGO group, which indicated MBGAPO exhibited a slightly stronger anti‐inflammatory effect than MBGO. This might be due to the antibacterial property of MBGAPO and the low immunogenicity of MBGAPO. All results demonstrated that MBGAPO established a low‐inflammation environment which was beneficial to bone regeneration.

### 2.5 Dynamic Immunomodulatary Effect of MBGAPO

**Figure 5:**
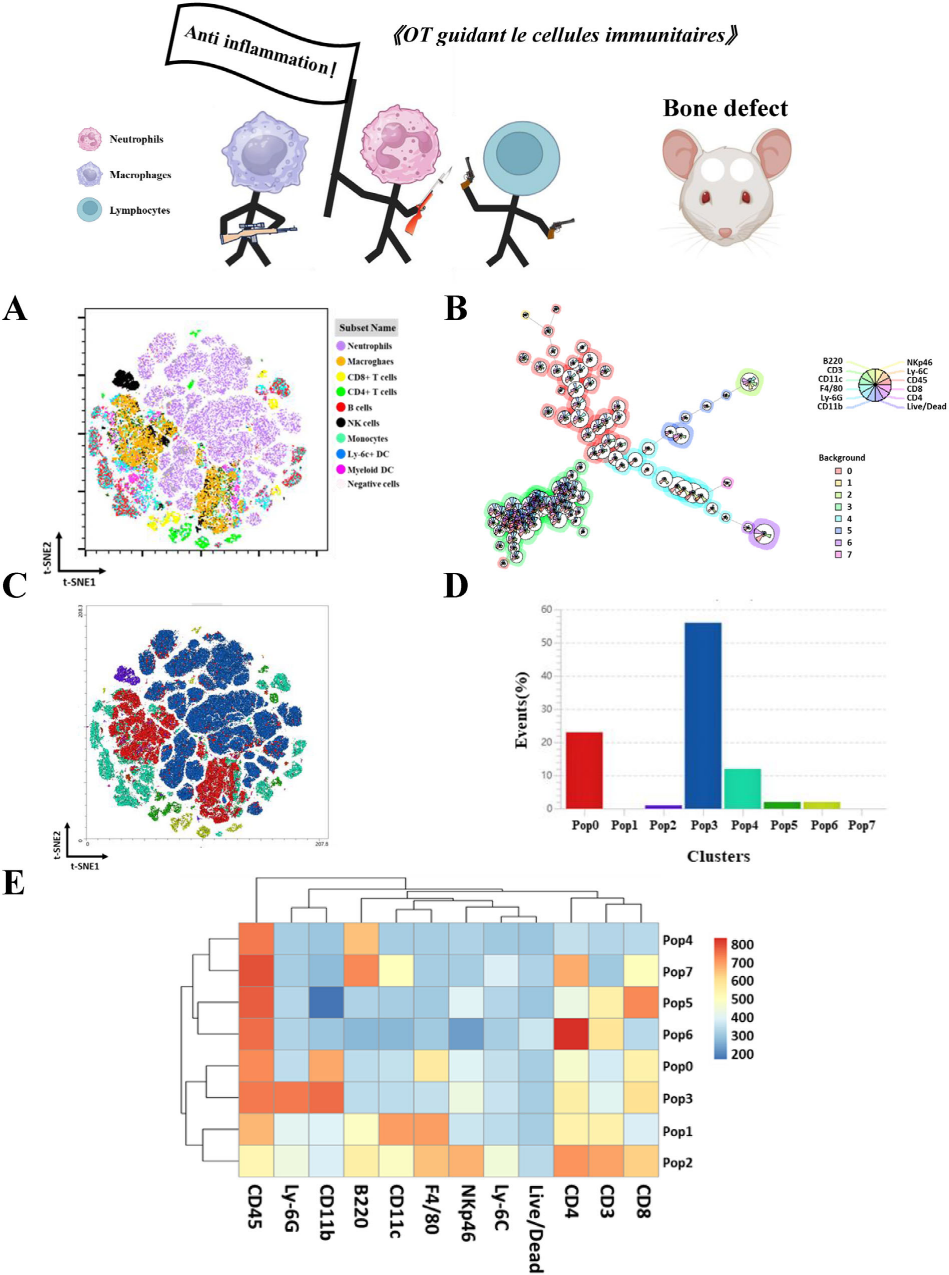
Immune cell heterogeneity in the bone regenerative microenvironment. A) T‐SNE analysis of the infiltrating immune cells. B) Clustering analysis of immune cells in pop0‐pop7. C) T‐SNE analysis of pop0‐pop7. D) Proportion of pop0‐pop7. E) Heatmap of immunologic markers expression levels in pop0‐pop7.

**Figure 6:**
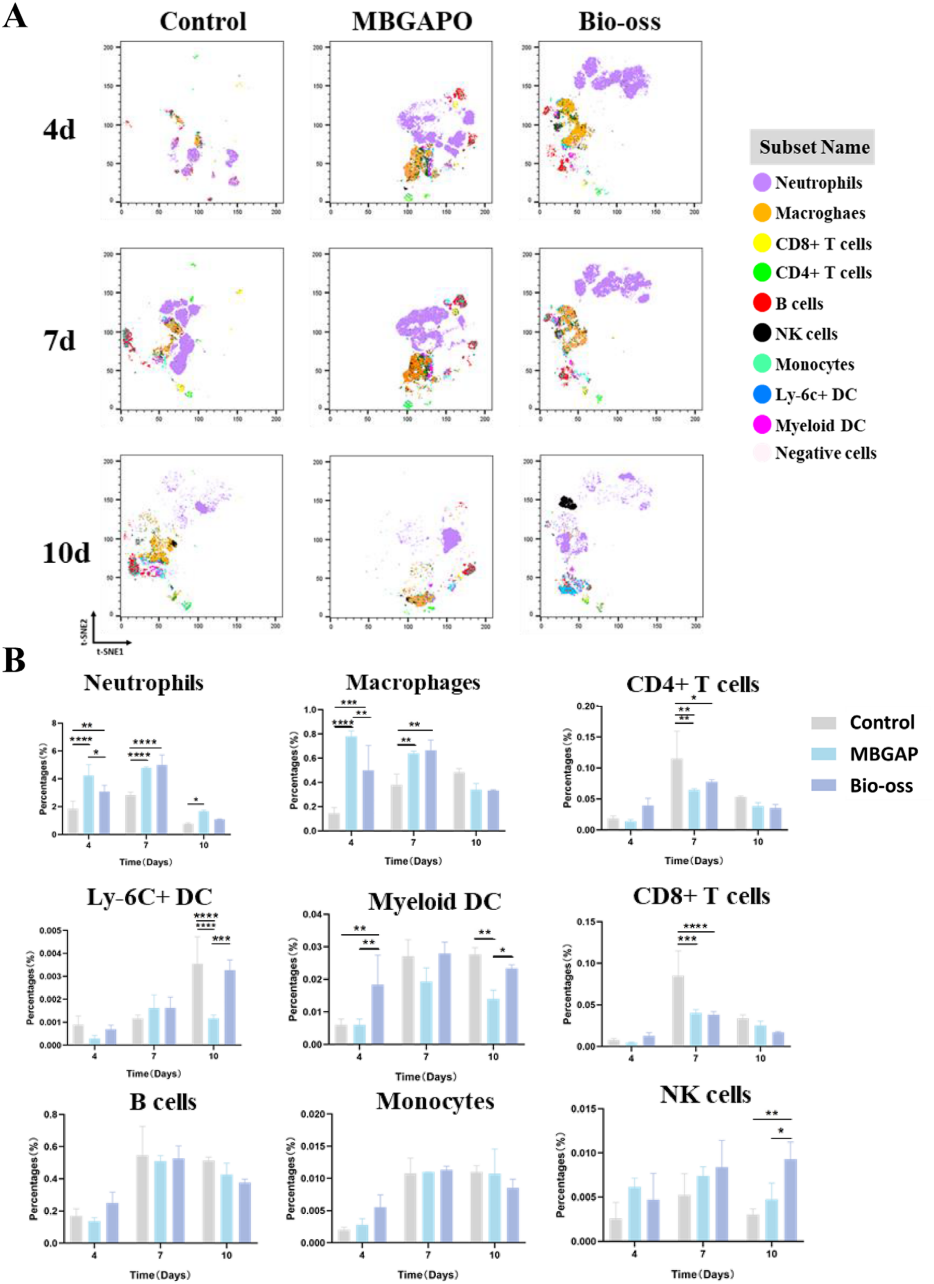
Distinct temporal immune cell constitutions of different groups at day 4, 7, 10. A) T‐SNE analysis of the infiltrating immune cells of different groups. B) Proportions of infiltrating immune cells of different groups. Bars The bars show the mean ± SD. ^*^ represents *p* < 0.05, ^**^ represents *p* < 0.01, ^***^represents *p *< 0.001, ^****^represents *p* < 0.0001. (*n* = 3).

Taken together, the above‐demonstrated excellent anti‐inflammatory effect of MBGAPO, flow cytometry of immune cells within the regenerative microenvironment was applied to identify the immune cell subgroup distribution. Bio‐oss is the most widely used bone substitute worldwide and is applied in minor augmentation, sinus floor elevation, and peri‐implantitis treatment.^[^ ^36^, ^37^, ^38^ ^]^ In order to characterize the immunologic diversity specifically within the microenvironment, MBGAPO and Bio‐oss were respectively implanted in a mouse cranial defect model. In our previous study, infiltrating immune cells underwent a typical alteration during day 4 to 10 and neutrophils and macrophages reached their peaks at day 7. Therefore, a 13‐color multiplex flow cytometry on infiltrating immune cells was performed at day 4, day 7, and day 10 after implantation. Major immune cells, including CD11b^+^Ly‐6G^+^ neutrophils, CD11b^+^F4/80^+^ macrophages, CD3^+^/ CD4^+^/CD8^+^ T lymphocytes, B220^+^ B cells, NKp46^+^ NK cells, and CD11c^+^dendritic cells (DCs) were identified (Figure S3, Supporting Information) in a t‐distributed stochastic neighbor embedding (t‐SNE) map of all infiltrating immune cells using the T‐SNE Plugin for Flowjo (**Figure** **5**A). With automated cluster explorer analyses, cells were divided into 8 clusters from pop0 to pop7 (Figure 5B) and presented in the same T‐SNE map as Figure 5A (Figure 5C). Cell number percentage distributions were shown in Figure 5D. Subgroups of immune cells divided by subjective gating and using the Cluster Explorer Algorithm exhibited great coincidence, which indicated that there was no subjective bias. Heatmap results demonstrated the expression pattern of pop0 to pop7 (Figure 5E). Pop3, pop4, pop5, and pop6 perfectly corresponded with neutrophils, B cells, CD8^+^ T cells, and CD4^+^ T cells. Pop0 were CD11b^+^ myeloid cells. Pop1 were F4/80^+^CD11c^+^ mononuclear phagocytes which were reported to exhibit high phagocytosis but low antigen‐presenting capacity.^[^ ^39^ ^]^ Pop2 and pop 4 represented a mixed cell group of T cells and NK cells, CD4+ T cells and B cells, respectively (**Figure** **6**A). All these results provided a landscape of distinct immune cell distribution in the regenerative microenvironment.

To further compare the immunologic niches among the MBGO group, Bio‐oss group, and the control group, we divided the whole t‐SNE map of Figure 5A on the basis of different groups and different time points (Figure 6B). Our results showed that neutrophils contributed to the most dominant infiltrating immune cells in the early stage of tissue regeneration, which reached the peak around 7 days and gradually decreased after 7 days (Figure 6B). Meantime, more neutrophils were summoned in the microenvironment in the MBGAPO group than other groups. Since neutrophils obtain energy from anaerobic glycolysis, they can survive in the local swelling, hypoxic, and poor‐blood‐flow environment after tissue injury.^[^ ^40^, ^41^, ^42^, ^43^, ^44^ ^]^ This may be the reason why they can be recruited into the regenerative microenvironment before macrophages. Due to the cytokine levels in the microenvironment, neutrophils around MBGAPO were mostly N2‐polarized, which exhibited an anti‐inflammatory phenotype (Figure 4C). In our study, the trend of the proportion of macrophages was consistent with that of neutrophils (Figure 6B, Neutrophils and Macrophages). The proportion of macrophages in the MBGAPO group was higher than other groups at day 4. At day 7, macrophage distribution of the Bio‐oss group surged to near the level of the MBGAPO group. It was reported that macrophages regulated bone regeneration through phagocytosis and clearance, cytokine secretion, regulation of osteoblasts and osteoclasts, participating in angiogenesis and immunomodulation.^[^ ^45^, ^46^, ^47^, ^48^ ^]^ Similar to neutrophils, according to the results of cytokine expression, macrophages around MBGAPO were mostly M2‐polarized macrophages, which exhibited anti‐inflammatory effect and promoted bone regeneration. More infiltrating neutrophils and macrophages in the MBGAPO group were summoned and earlier entered into the immune microenvironment (IME) than other groups, quickly reached a peak around day 7, then gradually reduced. This indicated that MBGAPO recruited N2‐polarized neutrophils and M2‐polarized macrophages in the early stage of bone regeneration. MBGAPO not only quickly triggered the inflammation response but also alleviated the inflammation. As a result, a low‐inflammatory IME was created, which was beneficial to bone regeneration.

It was demonstrated that CD4^+^ T cells did not show significant differences among the three groups, except at day 7 (Figure 6B). The proportion of CD4^+^ T cells was higher in the control group than in the other two groups at day 7. However, due to the high expression of TNF‐α and IL‐1, these CD4^+^ T cells were more likely to be Th1 cells, which exerted negative effects on bone regeneration.^[^ ^49^ ^]^ It was demonstrated that there were fewer myeloid dendritic cells (DCs), which were a major DC subgroup, and fewer Ly‐6C^+^ DCs in the MBGAPO group (Figure 6B). This may result from the low immunogenicity of MBGAPO through alleviating local inflammation. Recruiting of CD8^+^ T cells relied on the antigen‐presenting effect of DCs, the proportion of CD8^+^ T cells in the MBGAPO group was lower than in the other groups (Figure 6B). In our study, DCs increased at a later time, partly explaining the late increase of CD8^+^ T cells. These results demonstrated that MBGAPO had a low immunogenicity and triggered a diminished adaptive immune response.

Monocytes have also been reported to have a role in promoting bone regeneration.^[^ ^50^ ^]^ In our study, monocytes did not show a significant difference (Figure 6B). However, our gating method only marked a subset of cells equivalent to human classical/intermediate monocytes (Figure S2, Supporting Information). This result may not be fully representative of all monocytes. B cells played an important role in pathogen clearance ^[^ ^51^ ^]^ and did not show significant differences in our study (Figure 6B). NK cells are an important component of innate immunity. They have a killing effect on pathogens and infections, and have been reported to induce inflammation in rheumatoid arthritis (RA). Notably, it was also found in our study that B cells, monocytes, and NK cells did not show significance among the three groups and comprised only tiny proportions, which indicated they may not be the pivotal cells in the early stage of bone regeneration (Figure 6B).

## 3 Discussion

In our study, it was shown that MBGAPO significantly increased neutrophil and macrophage infiltrations at the early stage of bone regeneration (Figure 6B), which were crucial to the regeneration. Some studies have reported that neutrophils not only enter the regenerative microenvironment before macrophages, but also are the key cells to initiate endogenous bone regeneration and determine the outcome of regeneration.^[^ ^52^, ^53^ ^]^ Both of them were regarded as the most pivotal cells to regulate inflammation and indirectly affect bone regeneration. In our study, it was demonstrated that neutrophils and macrophages were the predominant immune cells during the early stage of bone regeneration, MBGAPO facilitated neutrophil and macrophage infiltration earlier into the regenerative microenvironment and exhibited an anti‐inflammatory effect. The expression levels of pro‐inflammatory cytokines such as IL‐1β and TNF‐α in the regenerative microenvironment at day 7 after scaffold implantation were lower than those in the control group (Figure 4C). This indicated that most of the neutrophils and macrophages in the regenerative microenvironment were in the N2‐polarized and M2‐polarized states. This validated that MBGAPO not only initiated the immune response more quickly, but also created a low‐inflammation niche. Previous studies have shown that the initial polarization state of neutrophils determines the fate of regeneration.^[^ ^52^ ^]^ This is also a hint to us that regulating the initial polarization state of neutrophils in the early stage has important meaning for tissue regeneration. It was reported that neutrophils promoted bone regeneration probably through secreting CXCL12, which could bind to the CXCR3 receptor on BMSCs to recruit BMSCs into the regenerative microenvironment.^[^ ^53^ ^]^ Bioactive scaffolds combined with engineered neutrophils/macrophages could be a promising therapeutic strategy for bone defects.

In addition to quantities, functions, and states of activation of immune cells were also important to be evaluated. Inflammation played a role in bone regeneration and was regulated by diverse immune cells and related cytokines. It was widely reported that N2‐polarized neutrophils and M2‐polarized macrophages exhibited a function of inhibiting inflammation and promoting bone regeneration. In our study, we found that MBGAPO also improved the state of local inflammation with downregulated pro‐inflammatory cytokines and upregulated anti‐inflammatory cytokines. Our next priority will be identifying and modifying different subgroups of neutrophils and macrophages in the regenerative microenvironment. In future research, high‐dimensional bulk sequencing and single‐cell sequencing should be applied to observe alternations at the genetic level and find new functional subgroup immune cell. Immune cells with different activation states play completely different roles during bone regeneration. Therefore, several functional markers of immune cells will be tested to identify the state of activation.

**Scheme 1:**
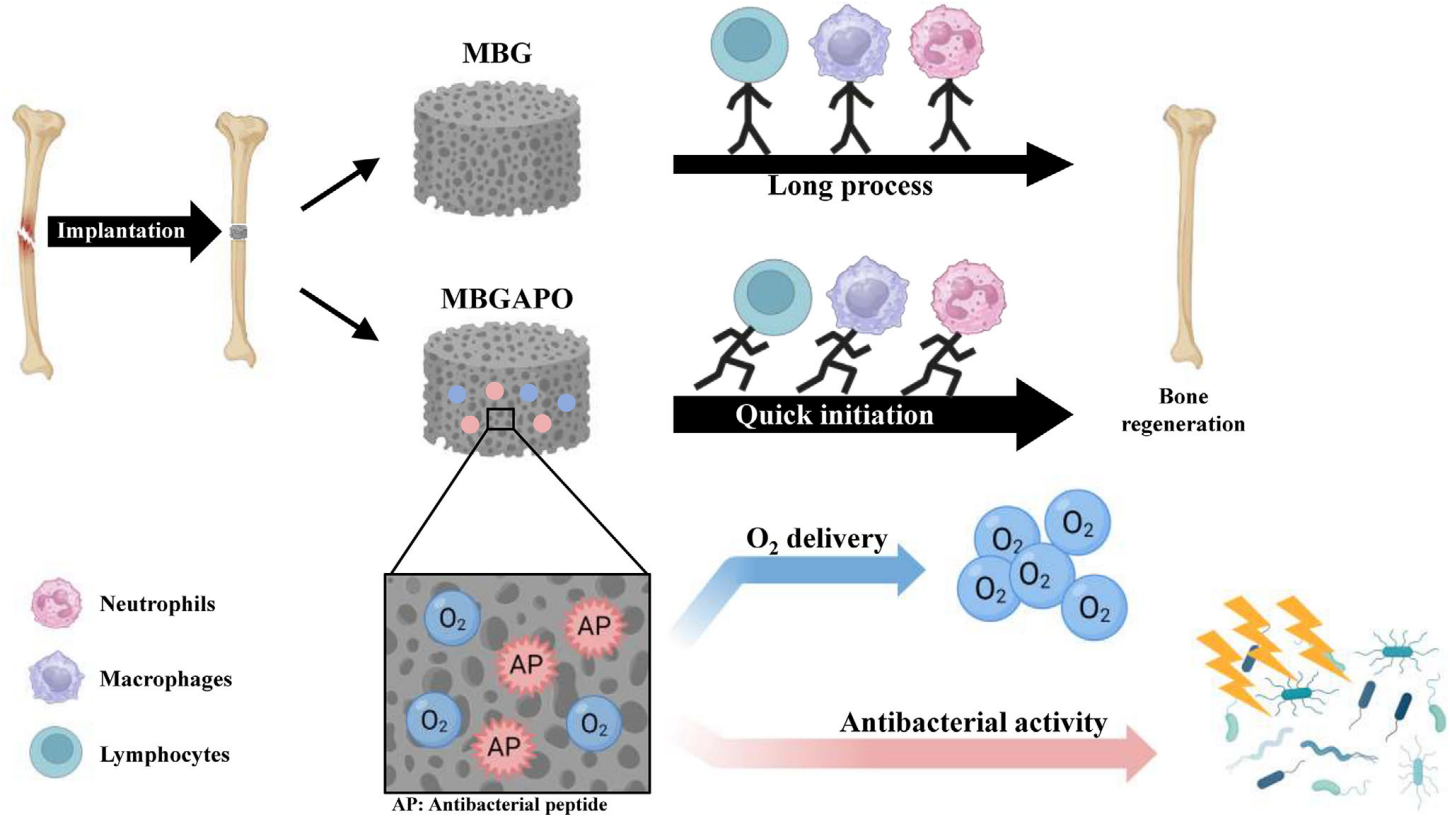
Graphic abstract of the MBGAPO scaffold.

Altogether, a multiplex‐immunomodulatory MBGAPO scaffold was synthesized, which first used a mesoporous structure as an oxygen‐storing and delivering carrier. As illustrated in the **Scheme** **1**, MBGAPO established a low‐inflammation environment and a more suitable regeneration niche for bone regeneration than the most widely used bone substitute Bio‐oss. It was verified that MBGAPO had promising antibacterial ability and oxygen‐releasing capacity. MBGAPO also exhibited good osteogenesis‐inducing function both in vitro and in vivo. MBGAPO provides a new regeneration solution for bone defect clinical treatment. MBGAPO could be prospectively studied as a promising osteoimmunology therapeutic strategy for clinical translation in the near future, which could be extended to other large multiple bone fracture applications and arouse broader interests of researchers.

## 4 Conclusion

In our study, we fabricated an MBGAPO scaffold with oxygen delivery in mesopores and antibacterial activity endowed by an antimicrobial peptide. MBGAPO exhibited sufficient oxygen‐storing and releasing capacity, promising antibacterial ability against *E. coli* and *MRSA*, and promoted osteogenesis both in vitro and in vivo. In cranial defect models, MBG and MBGAPO showed immunomodulatory effects that facilitated bone regeneration. Via systematically investigating the alternations of immune niches influenced by MBGAPO and compared with commercial Bio‐oss bone substitute, it was demonstrated that MBGAPO significantly increased neutrophil and macrophage infiltration at the early stage of bone repair, and created a milder (lower‐inflammatory) immune microenvironment by downregulating pro‐inflammatory cytokines and upregulating anti‐inflammatory cytokines secretion. In conclusion, MBGAPO provided a novel regenerative solution for clinical treatment of bone defects by combining oxygen therapy and an antimicrobial peptide. The mesoporosity‐based oxygen delivery has first been proven as a promising osteoimmunology therapeutic strategy, and its combination with antimicrobial peptides could be extended to other regenerative and disease treatment applications and arouse broader interests of researchers.

## 5 Experimental Section

### Materials

Antimicrobial peptide (Figure S1, Supporting Information) was synthesized by Allpeptide Biotechnology Co., Ltd (Hangzhou, China) and stored at − 20 ◦C.^[^ ^54^ ^]^ Dissolved oxygen meter was from Smart Sensor (AR8406, China). LIVE/DEAD bacterial staining kit was from Beyotime (C2030S, China). CCK8 Kit was from Dojindo (CK04, Japan). Fetal bovine serum (abs972), paraformaldehyde (abs9179), BCIP/NBT alkaline phosphatase (ALP) color development kit (abs9332), and alizarin red S (ARS, abs42012987) were from Absin (China). OriCell rat bone marrow mesenchymal stem cell osteogenic induction differentiation kit (RAXMX‐90021) was from Oricellbio. Evo M‐MLV One Step RT‐qPCR Kit II (AG11713) was from ACCURATE BIOTECHNOLOGY (HUNAN）CO., LTD（ChangSha， China）. LEGENDplex Rat Inflammation Panel, including IL‐33, IL‐1α, IFN‐γ, TNF‐α, CCL2 (MCP‐1), IL‐12p70, IL‐1β, IL‐10, IL‐6, IL‐18, IL‐17A, CXCL1 (KC), GM‐CSF was from Biolegend (741 396, USA). IV collagenase, hyaluronidase, and DNAase I were from Sigma (USA). Bio‐oss was purchased from Geistlich Pharma AG (Switzerland). All antibodies for flow cytometry were from BD (USA).

### Scaffold Preparation, Antimicrobial Peptide Functionalization, and O_2_ Loading

MBG scaffolds were fabricated as previously reported via a modified multi‐template method with F127 as mesoporous template and polyurethane sponge as macroporous template.^[^ ^55^, ^56^ ^]^ Briefly, 8 g of F127, 0.92 g of triethyl phosphate, 4 g of HCl (1 m), 20.8 g of Tetraethyl orthosilicate, and 3.08 g of Ca (NO3)2⋅4H2O were dissolved in 200 g of ethanol and stirred at 40 °C for 24 h. After sterilization, 1 mg mL^−1^ of antimicrobial peptide (AP) was added onto MBG scaffold (≈50 mg) at a saturated adsorption volume (40 µL), left at 4 °C overnight, and lyophilized to achieve an AP‐functionalized scaffold (MBGAP). MBG and MBGAP scaffolds were placed in a sterile chamber, vacuumized to eliminate the air in mesopores, and then inflated with O_2_ (flow rate = 5 L min^−1^) for 1 min to complete O_2_ loading to fabricate MBGO and MBGAPO scaffolds, respectively. All scaffolds were stored in air‐tight containers before use.

### Scaffold Characterization

Micro‐scale morphology and mesoporous structure of scaffolds were observed by scanning electron microscopy (SEM, ZEISS, GeminiSEM 300, Germany) and transmission electron microscope (TEM, HITACHI, H‐7650, Japan). Wide‐angle X‐ray diffraction (XRD, Panalytical, X'Pert PRO MPD, Netherlands) was applied for phase composition characterization, and energy dispersive spectrum (EDS) for element composition analyses.

### O_2_ Release Measurement

O_2_ release of scaffolds was measured by a dissolved oxygen meter at room temperature. According to the manufacturer's instructions, with the oxygen probe inserted, the scaffolds were immersed in the ddH_2_O (60 mg scaffold/1 mL ddH_2_O). Results were recorded after the readings were stable. At each time point before measurement, the probe was calibrated, and pure ddH_2_O was used as a negative control.

### Antibacterial Activity Assay

*Escherichia coli (E.coli)* and *Methicillin‐resistant Staphylococcus aureus (MRSA)* were used to detect the antibacterial capacity of scaffolds. Inhibition zone assay was performed as previously described.^[^ ^57^, ^58^ ^]^ Briefly, bacterial solution (3 × 10^8^ /mL) was dipped with a sterilized cotton swab and evenly seeded on an agar plate. Each scaffold (Ø5 mm) was placed on the plate and incubated at 37 °C for 6 h to observe the inhibition zone around the scaffold. Bacteria live/dead staining was performed using LIVE/DEAD Bacterial Staining Kit. Briefly, each scaffold (≈50 mg) was incubated with 1.5 mL bacteria (1 × 10^7^ mL^−1^) at 37 °C for 2 h. The scaffold was discarded, and bacteria were incubated with live/dead staining solution at room temperature for 1 h. After being washed with 0.85% NaCl twice, bacteria were resuspended and observed with a confocal fluorescence microscope (SP8, Leica, Germany). A blank group was set as the control.

### BMSC Extraction and CCK8 Assay

Rat bone marrow mesenchymal stem cells (BMSCs) were extracted from the rat femur as previously reported.^[^ ^55^ ^]^ Passage 3 (P3) BMSCs were used for further studies. BMSCs were seeded into a 96‐well plate (2000 cells per well) with scaffolds placed at the bottom of the well (≈15 mg per well). CCK8 assay was performed on days 1, 4, and 7 with CCK8 solution added into each well and incubated at 37 °C for 2 h. 450 nm OD values were measured using a spectrophotometer (Thermo Fisher, USA). A blank group was set as the control.

### ALP and ARS Staining

BMSCs (5 × 10^4^ cells per well) were co‐cultured with scaffolds (60 mg per well) in a 6‐well plate. Osteogenic inductive medium was added in (2 mL per well) and refreshed every 3 days. After 7, 14, and 21 days, cells were fixed with 4% paraformaldehyde for 15 min. Cells were stained with BCIP/NBT ALP color development kit or alizarin red S for 20 min and washed with PBS twice. The stained cells were observed with an inverted microscope (ZEISS, Germany). A blank group was set as the control.

### Real‐Time PCR

BMSCs (5 × 10^4^ cells per well) were co‐cultured with scaffolds (60 mg/well) in 6‐well plates for 7 or 14 days. RNA of cells was extracted using the Evo M‐MLV One Step RT‐qPCR Kit II. Expression of bone‐related genes was detected by qRT‐PCR using LightCycler 480 II (Roche, USA). Prime sequences were listed in Table S1 (Supporting Information). The mRNA expressions were calculated by the 2^‑ΔΔCt^ method.^[^ ^59^ ^]^ A blank group was set as the control.

### Animal Experiments

All animal experiments were approved by the Ethical Committee and performed following the guidelines of the Institutional Animal Care and Use Committees of Shanghai Jiao Tong University School of Medicine.

6‐week‐old female SD rats were applied to establish a full‐thickness cranial defect model. Briefly, the animals were anaesthetized with pentobarbitone. Skin and periosteum were incised, and the skull was exposed. A 5 mm‐diameter trephine bur was used for establishing the cranial defect model (bilateral). MBG, MBGO, or MBGAPO scaffolds were implanted into the defect, and the skin and periosteum were sutured layer by layer. After 7 days, the rats were euthanized, and the defect site was flushed with saline to achieve the early‐stage regenerative tissue specimens. After 8 and 12 weeks, the rats were euthanized to obtain the regenerated bone specimens. A blank group was set as the control.

Full‐thickness cranial defect model (2.5 mm diameter, bilateral) in 8‐week‐old female C57BL/6 mice was established for scaffold implantation. The mice were euthanized after 4, 7, and 10 days, and the defect site was flushed with saline to achieve the early‐stage regenerative tissue specimens. A blank group was set as the control.

### Micro‐CT Analysis

The regenerated bone specimens of rats were analyzed with microcomputed tomography (micro‐CT; PerkinElmer Quantum GX, USA) to evaluate the bone formation in the defect area. The bone defect areas were selected as the region of interest (ROI). Bone volume/total volume (BV/TV) and trabecular thickness (Tb.Th.) were analyzed and quantified with the software Analyze 12.0 (PerkinElmer).

### Multiple Cytokine Analysis

The early‐stage regenerative tissues of the rat were lysed following the previously reported protocol.^[^ ^60^ ^]^ Then cytokine levels in the lysates were evaluated with LEGENDplex Rat Inflammation Panel. This panel included most main inflammation‐related cytokines secreted by immune cells (IL‐1α, IL‐1β, IL‐6, IL‐10, IL‐12p70, IL‐17A, IL‐18, IL‐33, CXCL1, CCL2, IFN‐γ, TNF‐α, and GM‐CSF). Data were analyzed by LEGENDplex Data Analysis Software Suite‐Qognit online (https://legendplex.qognit.com/).

### Multiple Flow Cytometry

The early‐stage regenerative tissues of mice were ground and digested with a mixed enzyme cocktail (50 IU mL^−1^ IV collagenase, 280 IU mL^−1^ hyaluronidase, and 30 IU mL^−1^ DNAase I) for 1 h, washed twice with PBS, and resuspended 1 × 10^6^ cells/tube for flow cytometry analysis. The cells were incubated with antibodies (Table S2, Supporting Information) at 4 °C overnight, washed with PBS, resuspended, and analyzed by BD Fortessa X‐20 Flow cytometry analyzer (BD, USA). Data was analyzed by FlowJo 10.9.0. Automated cluster explorer analyses were applied using the Cluster Explorer Plugin of Flowjo.

### Statistical Analysis

All data in this study are presented as mean ± SD. Two‐way ANOVA nonparametric followed by Tukey's post hoc analysis was performed to identify significant differences. GraphPad Prism 8 software was used for all statistical analyses. *p* < 0.05 was considered a statistically significant difference.

## Conflict of Interest

The authors declare no conflict of interest.

## Supporting information



Supporting Information

## Data Availability

The data that support the findings of this study are available from the corresponding author upon reasonable request.
