# Supplementary material for: A “Mesoporous Oxygen Chamber” Scaffold with Antibacterial and Early Immunomodulatory Effect for Promoting Bone Regeneration
Source: Adv Sci (Weinh). 2025 Sep 16;12(41):e06737. doi: 10.1002/advs.202506737 (PMC12591200; doi:10.1002/advs.202506737)
Supplement: Supplementary file 1 — Supporting Information [file ADVS-12-e06737-s001.docx]

**A “Mesoporous Oxygen Chamber” Scaffold with Antibacterial and Early Immunomodulatory Effect for Promoting Bone Regeneration**

You Fu^1*^, Dan Lin^2*^, Zhicen Lu^3*^, Jian Wang^5^, Jing Zhao^6^, Zhiyuan Zhang^7^, Bing Fang^1#^ and Xiao Yang^4#^

^1^ Department of Orthodontics, Shanghai Ninth People's Hospital, Shanghai Jiao Tong University School of Medicine; College of Stomatology, Shanghai Jiao Tong University; National Center for Stomatology; National Clinical Research Center for Oral Diseases; Shanghai Key Laboratory of Stomatology & Shanghai Research Institute of Stomatology; Research Unit of Oral and Maxillofacial Regenerative Medicine, Chinese Academy of Medical Sciences, Shanghai 200011, China.

^2^ Shanghai University of Medicine and Health Sciences, Shanghai 201318, China

^3^ Fujian Key Laboratory of Oral Diseases & Fujian Provincial Engineering Research Center of Oral Biomaterial & Stomatological Key Laboratory of Fujian College and University, School and Hospital of Stomatology, Fujian Medical University, Fuzhou 350122, China.

^4^ Department of Oral & Cranio-Maxillofacial Surgery, Shanghai Ninth People's Hospital, Shanghai Jiao Tong University School of Medicine; College of Stomatology, Shanghai Jiao Tong University; National Center for Stomatology; National Clinical Research Center for Oral Diseases; Shanghai Key Laboratory of Stomatology & Shanghai Research Institute of Stomatology; Research Unit of Oral and Maxillofacial Regenerative Medicine, Chinese Academy of Medical Sciences, Shanghai 200011, China.

^5^ Department of General Dentistry, Shanghai Ninth People's Hospital, Shanghai Jiao Tong University School of Medicine; College of Stomatology, Shanghai Jiao Tong University; National Center for Stomatology; National Clinical Research Center for Oral Diseases; Shanghai Key Laboratory of Stomatology & Shanghai Research Institute of Stomatology; Research Unit of Oral and Maxillofacial Regenerative Medicine, Chinese Academy of Medical Sciences, Shanghai 200011, China.

^6^ Shanghai Dingchang Labor Security Consulting Co., Ltd, Shanghai 200011, China.

^7^ Department of Oral and Maxillofacial-Head Neck Oncology, Shanghai Ninth People's Hospital, Shanghai Jiao Tong University School of Medicine, College of Stomatology, National Center for Stomatology, National Clinical Research Center for Oral Diseases, Shanghai Key Laboratory of Stomatology & Shanghai Research Institute of Stomatology; Research Unit of Oral and Maxillofacial Regenerative Medicine, Chinese Academy of Medical Sciences, Shanghai 200011, China.

* These authors contribute equally to the article.

# Corresponding Author: Xiao Yang and Bing Fang, Shanghai Ninth People's Hospital, Shanghai Jiao Tong University School of Medicine, No. 639, Zhizaoju Road, Shanghai 200011, China. Phone: 8602138452590; E-mail: yangxiao0532@hotmail.com and fangbing@sjtu.edu.cn.

**
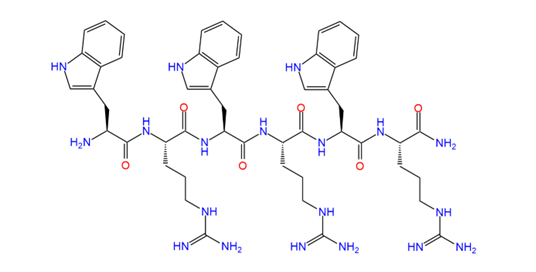
**

**Figure S1.** Chemical Structural Formula of the antimicrobial peptide.


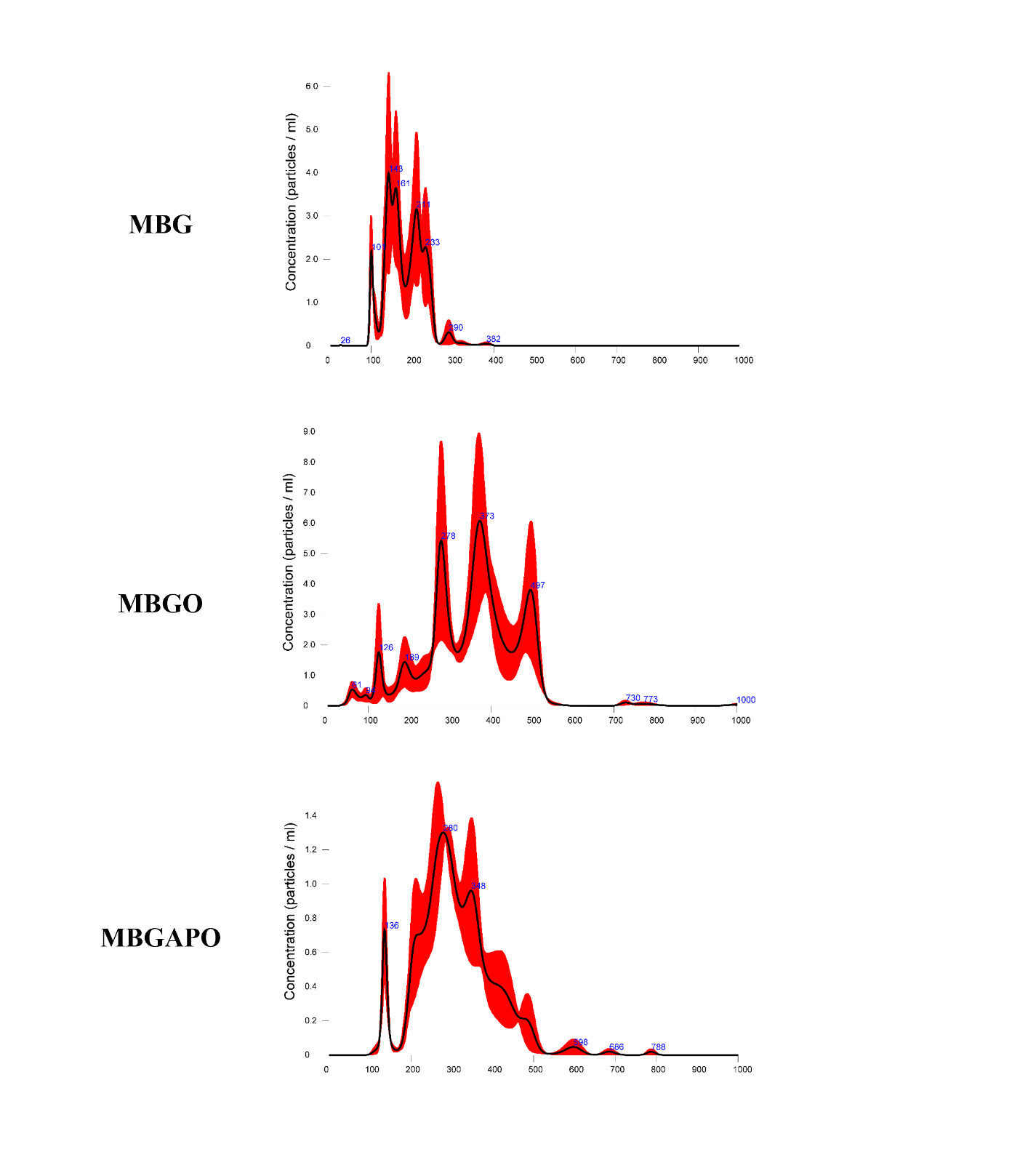


**Figure S2.** Pore sizes of MBG/MBGO/MBGAPO scaffolds.

**
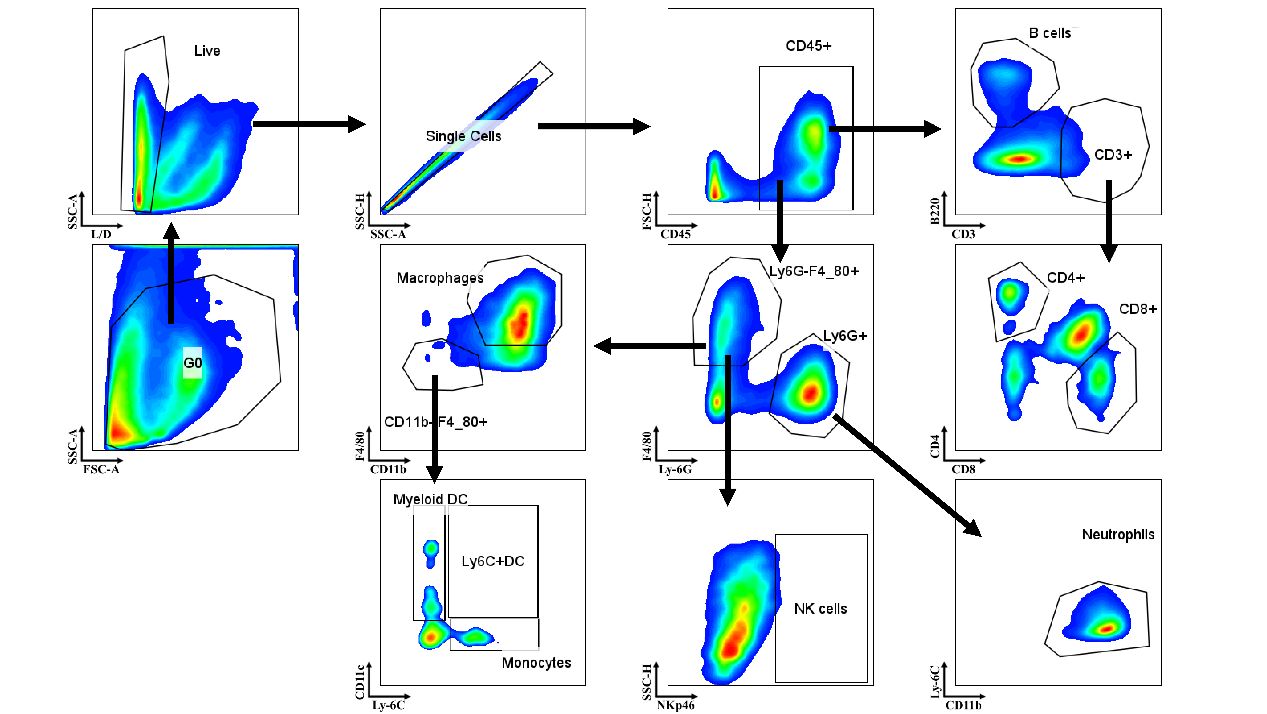
**

**Figure S3.** Gating strategy of multiplex flow cytometry analysis.

**
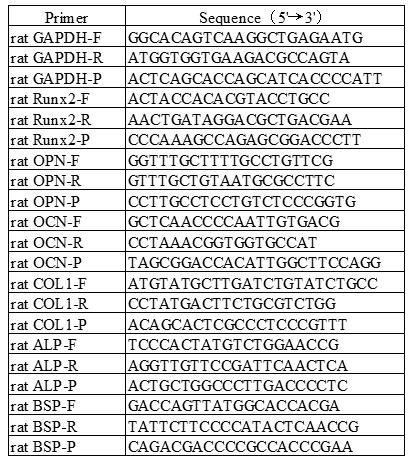
**

**Table S1.** Prime sequences for real-time PCR.


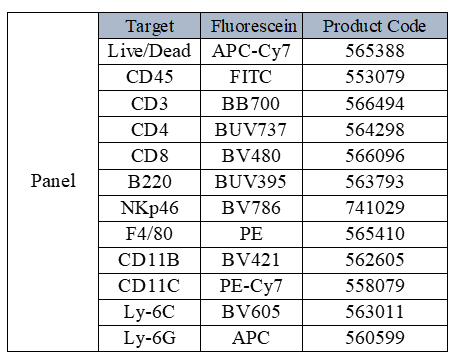


**Table S2.** Antibodies for multiplex flow cytometry.
